# Editorial: lncRNA in plants: function, mechanisms and applications

**DOI:** 10.3389/fpls.2023.1238185

**Published:** 2023-06-28

**Authors:** Jun Cui

**Affiliations:** Hunan Provincial Key Laboratory for Microbial Molecular Biology, State Key Laboratory of Developmental Biology of Freshwater Fish, College of Life Science, Hunan Normal University, Changsha, China

**Keywords:** lncRNA, function, mechanisms, lncRNA-encoded peptides, lncRNA targets

Up to 90% of genomes is estimated to be transcribed in eukaryotes, but only 2% of transcribed RNAs will code for proteins. The non-coding genome, also dubbed as the “dark matter”, includes a plethora of non-coding RNAs (ncRNAs) with unarguable biological functions such as the long non-coding RNAs (lncRNAs). The rapid development of omics sequencing technology has facilitated the identification of thousands of lncRNAs in plant species, but the role of lncRNAs in plants remains largely unexplored. This Research Topic encompasses two original research articles and two review articles.

Chen et al. provide an overview of the molecular mechanisms of lncRNAs in interacting with other biomolecules. LncRNAs function through interacting with RNAs, DNAs, proteins and peptide. Firstly, lncRNAs play multiple roles as an endogenous target mimic to repress miRNAs to regulate the expression of miRNA target genes, a precursor of phasiRNAs and miRNAs, and antisense transcripts co-expressed with mRNAs. Secondly, lncRNA–DNA binding modulates chromatin loop dynamics, gene transcriptions and DNA transcriptions. Thirdly, links between lncRNAs and proteins are lncRNAs as guides for recruiting proteins, decoys for hijacking proteins, and scaffolds for linking multiple proteins together. Last, in addition to interacting with proteins, lncRNAs interact with peptides and they can also code peptides. Similar mechanisms of interaction between plant lncRNA and other macromolecules can be found in eukaryotes such as humans, mammals, fungi, further suggesting the conservation of biological evolution on the earth.

Most lncRNAs are not conserved in different species, but it is found that lncCOBRA transcripts are identified for their high level of sequence conservation in the related crop species *Brassica rapa*. Among them, lncCOBRA1 is annotated to be a 318 nt lincRNA in the Araport11 genome annotation and contains two small nucleolar RNA (snoRNA) sequence domains within it. Given the evident importance of sno-lncRNAs in humans, these snoRNA sequences and orientation are highly conserved, suggesting they are of significant evolutionary importance. lncCOBRA1 displays high levels early in germination. Furthermore, the plants lacking lncCOBRA1 display patterns of delayed germination and are overall smaller than wild-type plants, as shown as low percent seeding with emerged cotyledons, low number of leaf primordia, small plant size and low fresh weight. To understand the molecular function of lncCOBRA1, 113 proteins are identified as high-confidence lncCOBRA1-interacting proteins. Of which, lncCOBRA1 may act as a scaffold with the RACK1A protein to regulate germination and development, possibly through a role in ribosome biogenesis. This suggest that lncCOBRA1 is a conserved lncRNA and plays an important role in germination and development by interaction with RACK1A protein as a scaffold (Kramer et al.).

In addition, other lncRNAs acted in bud development is also identified from tobacco plants. The hormone signal transduction and glycometabolism are related to the promoted growth of axillary bud by topping. In these pathways, the genes are trans-regulated by differentially expressed lncRNAs, such as MSTRG.52498.1, MSTRG.60026.1, MSTRG.17770.1, and MSTRG.32431.1. These lncRNAs are related to decreased auxin, abscisic acid and gibberellin, and increased cytokinin. MSTRG.28151.1 is identified as the antisense lncRNA of *NtTB1* (increased axillary buds). The tobacco plants silencing MSTRG.28151.1 display the decreased expression of *NtTB1* and larger axillary buds, suggesting the vital function of MSTRG.28151.1 axillary bud development by *NtTB1*. This suggesting that lncRNA-mRNA interactions also affect germination and development, besides lncRNAs interesting with proteins (Wang et al.).

Recently, an interesting phenomenon that ncRNAs have the ability to encode small peptides through their small open reading frames (sORFs) is discovered. Sruthi et al. review the emerging roles of sORFs and small peptides encoded by lncRNA, circRNAs, and precursor miRNAs in plants. The evolutionary role of smORFs in non-coding RNAs is briefly discussed, with an overview of various methods used for their identification. The small peptides coded by ncRNAs are involved in plant growth and development, regulation of programmed cell death, plant and pathogen interaction and others. The different methods are being employed to identify the small peptides encoded by the ncRNAs. Bioinformatics has been used to identify the small peptides encoded by the ncRNAs and analyze their function, including FuncPEP, SmProt, PsORF and others. Mass spectrometry comparison, western blotting, initiation codon activity analysis, omics analysis and other experimental validations are helped to determine whether the ORFs are translated and functional in plant. Combining multiple methods could help to increase the accuracy of detection. Small peptides are identified using bioinformatics datasets and tools, and then validated using transcriptome, proteome analysis and biological experiments.

In summary, this Research Topic features diverse research efforts and methodologies aimed at putting the spotlight on lncRNA to gain more perspective on their mechanisms and functions. These studies review the molecular mechanisms of lncRNAs in interacting with other biomolecules and the emerging roles of sORFs and small peptides encoded by ncRNAs, provide the novel mechanisms of lncRNA action in which it may act as a scaffold with the target protein and as the antisense lncRNA of target gene to regulate growth and development. The knowledge gained from this Research Topic has the potential to pave the way for improving and perfecting the molecular mechanism of lncRNA action and using lncRNAs as the candidates for plant breeding.

## Author contributions

The author confirms being the sole contributor of this work and has approved it for publication.

